# Crystal engineering in **IUCrJ** 2021: interactions, structures, properties

**DOI:** 10.1107/S2052252522004274

**Published:** 2022-04-27

**Authors:** Gautam R. Desiraju

**Affiliations:** a Solid State and Structural Chemistry Unit, Indian Institute of Science, Bangalore, 560 012, India

**Keywords:** intermolecular interactions, crystal structure, crystallization

## Abstract

The articles in the chemistry and crystal engineering section of **IUCrJ** published from 2021 onwards continue to deal with themes like *interactions*, *structure* and *properties* taken in the broadest sense.

In 1989, I wrote that crystal engineering is the understanding of intermolecular interactions in the context of crystal packing and the utilization of such understanding in the design of new solids with desired physical and chemical properties (Desiraju, 1989 ▸). It is interesting to note that just about every one of the 25 odd papers in the chemistry and crystal engineering section of **IUCrJ** in 2021 continues to deal with themes like *interactions*, *structure* and *properties* taken in the broadest sense. There is increasing appreciation of the fact that a crystal structure is not a unique or distinguishing property of a molecule. A single molecule may have many crystal structures and some of these may be only stable under unusual temperature and pressure conditions. There may be others that only have an existence *in silico* at least at the present time. Papers have been published that deal with structures under extra-terrestrial conditions, under high pressures and elevated temperatures. Papers on polymorphs especially polymorphs of drugs continue to be popular as also are those that deal with clusters, which may be precursors for crystals for, in the end, the Holy Grail of crystal engineering is to find a general solution to the problem: *Given the molecular structure of a compound what are all the possible crystal structures?* The crystal engineering papers straddle conventional vertical classifications such as organic chemistry, inorganic chemistry, materials chemistry and nanochemistry indicating that these new ways of looking at traditional subjects might offer better and quicker solutions to problems related to complexity, emergence and other multivariate phenomena in the world of molecular crystals.

**IUCrJ** published 25 papers in the crystal engineering and chemistry section in 2021 out of the total of 190 in this section since the inception of the journal in 2014. **IUCrJ** published 110 papers in all sections last year as compared to the 760 that the journal has published in its entirety since its inception in 2014. The lower number might be due to the COVID pandemic. The chemistry component in 2021 is just about its running average across all years, but it must be noted that we have more sections now than we did in 2014. The papers in our section continue to contribute their share to reaching an impact factor of 4.769 in 2020. The contribution of this section has therefore stabilized while one would expect more papers in the MOF/COF area as also papers dealing with crystal structure determination using electron diffraction and charge density studies apart from the always popular area of pharmaceutical cocrystals and polymorphs including the events leading up to their crystallization.

*Papers published in chemistry and crystal engineering section of* ***IUCrJ** in 2021/2022*

*Hirshfeld atom refinement based on projector augmented wave densities with periodic boundary conditions*. P. N. Ruth, R. Herbst-Irmer & D. Stalke (2022). *IUCrJ*, **9**, 286–297.

*Formation and stabilization mechanism of mesoscale clusters in solution*. S. Zong, J. Wang, X. Huang, H. Wu, Q. Liu & H. Hao (2022). *IUCrJ*, **9**, 215–222.

*Crystallography relevant to Mars and Galilean icy moons: crystal behavior of kieserite-type monohydrate sulfates at extraterrestrial conditions down to 15 K*. M. Wildner, B. A. Zakharov, N. E. Bogdanov, D. Talla, E. V. Boldyreva & R. Miletich (2022). *IUCrJ*, **9**, 194–203.

*Insights into molecular recognition from the crystal structures of p-*tert*-butyl­calix[6]arene complexed with different solvents*. M. Malinska (2022). *IUCrJ*, **9**, 55–64.

*Stochastic hydration of a high-nitro­gen-content molecular compound recrystallized under pressure*. A. Olejniczak, A. Katrusiak, M. Podsiadło & A. Katrusiak (2022). *IUCrJ*, **9**, 49–54.

*Regioisomers of singly bridged calix[6]crown-6 and their heavy alkali metal complexes: a molecular baseball glove for caesium(I)*. S. Kim, J. H. Jung, S. S. Lee & I.-H. Park (2022). *IUCrJ*, **9**, 43–48.

*Differences in thermal expansion and motion ability for herringbone and face-to-face π-stacked solids*. X. Ding, E. Zahid, D. K. Unruh & K. M. Hutchins (2022). *IUCrJ*, **9**, 31–42.

*Using crystallography tools to improve vaccine formulations*. M. C. A. Fantini, C. L. P. Oliveira, J. L. S. Lopes, T. S. Martins, M. A. Akamatsu, A. G. Trezena, M. T.-D. - Franco, V. F. Botosso, O. A. B. E. Sant’Anna, N. Kardjilov, M. K. Rasmussen & H. N. Bordallo (2022). *IUCrJ*, **9**, 11–20.

*Accessing new polymorphs and solvates through solvothermal recrystallization*. D. R. Allan (2022). *IUCrJ*, **9**, 6–7.

*The benefits of Cu Kβ radiation for the single-crystal X-ray structure determination of crystalline sponges*. F. Meurer, C. von Essen, C. Kühn, H. Puschmann & M. Bodensteiner (2022). *IUCrJ*, **9**, 349–354.

*Effect of synthesis time on plasmonic properties of Ag dendritic nanoforests*. H. J. Huang, H.-W. Chang, C.-Y. Lee, M.-H. Shiao, Y.-L. Chiu, P.-Y. Lee & Y.-S. Lin (2022). *IUCrJ*, **9**, 364–369.

*Revealing the early stages of carbamazepine crystallization by cryoTEM and 3D electron diffraction*. E. T. Broadhurst, H. Xu, S. Parsons & F. Nudelman (2021). *IUCrJ*, **8**, 860–866.

*Quantifying magnetic anisotropy using X-ray and neutron diffraction*. E. A. Klahn, E. Damgaard-Møller, L. Krause, I. Kibalin, A. Gukasov, S. Tripathi, A. Swain, M. Shanmugam & J. Overgaard (2021). *IUCrJ*, **8**, 833–841.

*Template design based on molecular and crystal structure similarity to regulate conformational polymorphism nucleation: the case of α,ω-alkanedi­carb­oxy­lic acids*. J. Lin, P. Shi, Y. Wang, L. Wang, Y. Ma, F. Liu, S. Wu & J. Gong (2021). *IUCrJ*, **8**, 814–822.

*The mechanism driving a solid–solid phase transition in a biomacromolecular crystal*. S. Ramakrishnan, J. R. Stagno, W. F. Heinz, X. Zuo, P. Yu & Y.-X. Wang (2021). *IUCrJ*, **8**, 655–664.

*Charge density studies of multicentre two-electron bonding of an anion radical at non-ambient temperature and pressure*. V. Milašinović, K. Molčanov, A. Krawczuk, N. E. Bogdanov, B. A. Zakharov, E. V. Boldyreva, C. Jelsch & B. Kojić-Prodić (2021). *IUCrJ*, **8**, 644–654.

*Detection and characterization of folded-chain clusters in the structured melt of isotactic polypropyl­ene*. X. Li, J. Ding, P. Chen, K. Zheng, X. Zhang & X. Tian (2021). *IUCrJ*, **8**, 595–607.

*Distinct pathways of solid-to-solid phase transitions induced by defects: the case of DL-me­thio­nine*. G. Shi, S. Li, P. Shi, J. Gong, M. Zhang & W. Tang (2021). *IUCrJ*, **8**, 584–594.

*Structural chemistry of layered lead halide perovskites containing single octahedral layers*. J. A. McNulty & P. Lightfoot (2021). *IUCrJ*, **8**, 485–513.

*Unveiling the self-association and desolvation in crystal nucleation*. D. Li, Y. Wang, S. Zong, N. Wang, X. Li, Y. Dong, T. Wang, X. Huang & H. Hao (2021). *IUCrJ*, **8**, 468–479.

*Reduction of dislocations in α-Ga_2_O_3_ epilayers grown by halide vapor-phase epitaxy on a conical frustum-patterned sapphire substrate*. H. Son, Y. - Choi, S.-K. Hong, J.-H. Park & D.-W. Jeon (2021). *IUCrJ*, **8**, 462–467.

*Supramolecular structures of NiII and CuII with the sterically demanding Schiff base dyes driven by cooperative action of preagostic and other non-covalent interactions*. A. A. Shiryaev, T. M. Burkhanova, M. P. Mitoraj, M. Kukulka, F. Sagan, G. Mahmoudi, M. G. Babashkina, M. Bolte & D. A. Safin (2021). *IUCrJ*, **8**, 351–361.

*Electronic structure of Schiff-base peroxo{2,2′-[1,2-phenyl­enebis(nitrilo­methanylyl­idene)]bis­(6-meth­oxy­phenolato)}titanium(IV) monohydrate: a possible model structure of the reaction center for the theoretical study of hemoglobin*. J. A. Kožíšková, M. Breza, M. Valko, P. Herich, L. Bučinský & J. Kožíšek (2021). *IUCrJ*, **8**, 295–304.

*Coherent crystal branches: the impact of tetragonal symmetry on the 2D confined polymer nanostructure*. Z. Liang, N. Zheng, B. Ni, Z. Lai, H. Niu, S. Zhang & Y. Cao (2021). *IUCrJ*, **8**, 215–224.

*Nitro­sonium nitrate (NO^+^NO3^−^) structure solution using *in situ* single-crystal X-ray diffraction in a diamond anvil cell*. D. Laniel, B. Winkler, E. Koemets, T. Fedotenko, S. Chariton, V. Milman, K. Glazyrin, V. Prakapenka, L. Dubrovinsky & N. Dubrovinskaia (2021). *IUCrJ*, **8**, 208–214.

*Competitive cocrystallization and its application in the separation of flavonoids*. Y. Xia, Y. Wei, H. Chen, S. Qian, J. Zhang & Y. Gao (2021). *IUCrJ*, **8**, 195–207.

*Use of additives to regulate solute aggregation and direct conformational polymorph nucleation of pimelic acid*. P. Shi, S. Xu, H. Yang, S. Wu, W. Tang, J. Wang & J. Gong (2021). *IUCrJ*, **8**, 161–167.

*Increasing complexity: structural equivalence and combinatorial approaches to preparation of high order cocrystals*. S. A. Bourne (2021). *IUCrJ*, **8**, 152–153.
